# Bulk Versus Surface Modification of Alumina with Mn and Ce Based Oxides for CH_4_ Catalytic Combustion

**DOI:** 10.3390/ma12111771

**Published:** 2019-05-31

**Authors:** Stefan Neatu, Mihaela M. Trandafir, Adelina Stănoiu, Ovidiu G. Florea, Cristian E. Simion, Lucia N. Leonat, Cornel Cobianu, Marin Gheorghe, Mihaela Florea, Florentina Neatu

**Affiliations:** 1 National Institute of Materials Physics, Atomistilor 405A, 077125 Magurele, Romania; stefan.neatu@infim.ro (S.N.); mihaela.trandafir@infim.ro (M.M.T.); adelina.stanoiu@infim.ro (A.S.); ovidiu.florea@infim.ro (O.G.F.); simion@infim.ro (C.E.S.); lucia.leonat@infim.ro (L.N.L.); mihaela.florea@infim.ro (M.F.); 2 NANOM-MEMS SRL, G. Cosbuc 9, 505400 Rasnov, Romania; cornel.cobianu@gmail.com (C.C.); maringhe@nanom-mems.com (M.G.)

**Keywords:** surface vs bulk, mixed oxides, methane combustion

## Abstract

This study presents the synthesis and characterization of lanthanum-modified alumina supported cerium–manganese mixed oxides, which were prepared by three different methods (coprecipitation, impregnation and citrate-based sol-gel method) followed by calcination at 500 °C. The physicochemical properties of the synthesized materials were investigated by various characterization techniques, namely: nitrogen adsorption-desorption isotherms, X-ray diffraction (XRD), X-ray photoelectron spectroscopy (XPS), scanning electron microscopy (SEM) and H_2_–temperature programmed reduction (TPR). This experimental study demonstrated that the role of the catalytic surface is much more important than the bulk one. Indeed, the incipient impregnation of CeO_2_–MnO_x_ catalyst, supported on an optimized amount of 4 wt.% La_2_O_3_–Al_2_O_3,_ provided the best results of the catalytic combustion of methane on our catalytic micro-convertors. This is mainly due to: (i) the highest pore size dimensions according to the Brunauer-Emmett-Teller (BET) investigations, (ii) the highest amount of Mn^4+^ or/and Ce^4+^ on the surface as revealed by XPS, (iii) the presence of a mixed phase (Ce_2_MnO_6_) as shown by X-ray diffraction; and (iv) a higher reducibility of Mn^4+^ or/and Ce^4+^ species as displayed by H_2_–TPR and therefore more reactive oxygen species.

## 1. Introduction

Complete oxidation of carbon-based fossil fuel in industrial chemistry and the automotive industry based on internal combustion engines is one of the biggest scientific challenges, aiming to reduce polluting gases being released into the atmosphere [1] with short-term toxic effects on people’s health and long-term effects on the environment in terms of global warming [2], as well as efficient energy generation. Historically, in this direction of minimizing the toxic emissions to the atmosphere, a major breakthrough for automotive and petrochemical industry was the invention of the catalytic converter, which further oxidized fossil fuel combustion products on the surface of high specific area metal oxides modified with platinum group metals [3]. However, the efficiency of these converters is low during the cold-start regime of the engine, when the temperature of the exhaust gases from the vehicle is below 200 °C and thus unable to heat the catalyst enough to trigger the catalytic oxidation of those gases [1]. During this critical operation time, the diesel oxidation catalysts can emit up to 80% of CO gas, for example [4].

Under these considerations, where the aerobic oxidation of these fuels requires high activation energy for the reactions of the C(sp^3^)–H bonds with the atmosphere air [5], incomplete combustion of the fuel will release into the atmosphere not only CO_2_ and water but also CO, hydrocarbon (HCs) (like soot or particle matter with a size below 2.5 µm) and NO_x_, as it is in the case of diesel fuel oxidation [6]. From this point of view, the utilization of natural gas as a fuel was considered a better and apparently cleaner alternative to both gasoline and diesel fuel, due to the increased efficiency of the natural gas motors by about 10–20% and an apparently softer impact on the environment [7,8].

These relatively improved properties of natural gas fuel come from the fact that it is mainly composed of methane and its combustion generates less NO_x_. On the other hand, the CH_4_ molecule is chemically more stable than the CO molecule and therefore higher temperatures are required for complete catalytic oxidation in the catalytic converter [9]. The complete combustion of methane will provide CO_2_ and H_2_O as reaction products. However, in the case of the incomplete oxidation of natural gas fuel, the release into the atmosphere of the remaining unoxidized CH_4_ will counterbalance the above benefits, due to the high global warming potential (it is a measure of how much energy the emissions of 1 ton of a gas will absorb over a given period of time, relative to the emissions of 1 ton of carbon dioxide) of methane, which is about 21 to 36 higher (over 100 years) than that of CO_2_ considered as reference and this result is explained by the higher amount of energy absorbed by CH_4_ molecules with respect to the CO_2_ molecule [10,11].

The present catalysts used for the oxidation of the mentioned fossil fuel consist of palladium as an active phase supported on a high specific area metal oxide [12]. In such catalytic converters, the key metal oxide supporting the noble metal catalyst is CeO_2_ due to its oxygen store and release capabilities, multiple oxidation states of the cerium cations and their easy redox reactions (Ce^4+^/Ce^3+^), which promote oxygen vacancy formation [13]. Such Pd–CeO_2_ catalysts take advantage of the low cost Pd catalyst, high oxidation capability and better sintering resistance with respect to platinum and rhodium-based catalysts [4]. However, despite the improvements brought to these Pd-ceria based catalysts, there are still issues with complete oxidation of fossil fuel at low temperatures, for both diesel and natural gas, where CO and/or soot and/or CH_4_ are released into the atmosphere not only from the vehicles but also from coal mines (up to 1% CH_4_ in air) [14] and from different chemical processes in the petrochemical industry.

In the last two decades, intensive research has been pursued in the field of development of novel catalysts able to improve the efficiency and the reliability of the existing noble metal-based catalysts [15], as well as to create novel generations of noble metal-free catalysts, starting from the “traditional” ceria as a host, to which a great number of oxides of transition metals were added as possible dopants, as one of the starting approaches [16,17]. However, using pure bulk ceria as a catalyst was not a solution due to sintering processes, which occurred at higher temperatures, which further triggered the increase of the crystallite size and the reduction of the specific surface area, of oxygen vacancies number, as well as of catalytic properties [18]. Therefore, ceria-based composites, obtained by mixing with different metal oxides, became a clear option for the further improvement of these materials.

As a result of an extensive comparative study of the physical, morphological, structural and catalytical properties of noble metal-free ceria doped with cations of transition metals like manganese (Mn), zirconium (Zr), lanthanum (La), praseodymium (Pr), hafnium (Hf) and iron (Fe), very useful results were obtained, which allowed performance of a ranking of the catalytic properties of the ceria based composites with respect to low temperature combustion of carbon monoxide (CO) and soot. It was thus demonstrated that CeO_2_–MnO_x_ composites have shown the best catalytic behaviour; by showing that the temperature at which 50% of the CO or soot concentration in air was oxidized (T_50_) was lowest for CeO_2_–MnO_x_ in comparison with all the other mixed oxides. (T_50_ for CO was equal to 117 °C and T_50_ for soot was equal to 396 °C) [16].

These results for manganese-doped ceria were explained in terms of the lowest binding energy of lattice oxygen due to the major differences in the electronegativity of the Ce host and doping Mn cations, a maximum lattice strain enhancing the oxygen diffusion from bulk to the surface of the catalyst, as well as the multiple oxidation states of Mn in ceria.

An important experimental study by Venkataswamy et al. showed the catalytic activity during CO oxidation of the CeO_2_–MnO_x_ composite supported on pure alumina [19]. It was emphasized that for CO oxidation on CeO_2_–MnO_x_ composite calcinated at 500 °C and deposited on alumina substrate, the T_50_ was about 67 °C, which was much lower with respect to the T_50_ of pure ceria deposited on alumina or pure manganese oxide deposited on alumina, which were 304 °C and 374 °C, respectively. This result was excellent proof of the synergy of the two oxides in the composite formed between them. This study has also shown that the increase of calcination temperature of the alumina supported CeO_2_–MnO_x_ composite from 500 °C up to 800 °C has increased the T_50_ value up to 177 °C and such a result could be correlated to the structural changes in both the catalyst and pure alumina substrate.

Recently, an improved catalytic property of the mesoporous CeO_2_–MnO_x_ composite on low temperature oxidation of hydrocarbons has been demonstrated by Zhang et al. [5]. It was shown by these authors that dissolving and gelling of the two metal oxides precursors in the presence of a ionic liquid template followed by its extraction at 200 °C and thermal treatment at 500 °C has created a solid solution of CeO_2_–MnO_x_ with a high concentration of active oxygen, a lower energy of vacancy formation and high oxygen vacancy migration as an explanation of low temperature and selective oxidation of hydrocarbons (from cyclohexane to cyclohexanone), with T_50_ in the range of 100–120 °C and carbon monoxide with T_50_ at about 50 °C. In the same direction, Wang et al. have shown the role of the hydrothermal synthesis and optimization of the molar ratio of the Ce and Mn cations on the low temperature oxidation of benzene by the solid solution of CeO_2_–MnO_x_, with T_50_ at about 260 °C [20].

As can be inferred from the above studies, despite the high interest in the use of natural gas fuel instead of gasoline and Diesel fuel, due to its fewer toxic oxidation by-products, there are quite a few papers on the catalytic properties of the CeO_2_–MnO_x_ composite in the catalytic combustion of methane, as a main component of natural gas [21,22]. Thus, Shi et al. have shown the sensibility of the catalytic properties of CeO_2_–MnO_x_ composite to the effect of supporting substrate and the precursors used for the synthesis.

It is the purpose of our work to perform extended research on the catalytic performances of CeO_2_–MnO_x_ composite supported on lanthanum-modified alumina substrate as a function of three syntheses methods like coprecipitation, incipient impregnation and citrate based-sol gel, for methane combustion. For this study, for the incipient impregnation method we have used, for the first time, a commercial, thermally stable 4 wt.% La_2_O_3_–Al_2_O_3_ support for the distribution of the CeO_2_–MnO_x_ catalyst on the outer surfaces of support particles, while for the rest of the experiments, the precursors of the ceria and manganese oxide were mixed and precipitated simultaneously together with the precursors of the lanthanum oxide and alumina. Therefore, the role of bulk versus surface modification of alumina with Mn and Ce will be discussed.

Finally, for the evaluation of the catalytic properties of the above catalysts as a function of these process parameters, we have devised a self-heated catalytic micro-converter, consisting of an alumina strand above which a resistive platinum meander was deposited by thick film technology. Above this future heater, we have deposited the CeO_2_–MnO_x_ catalyst supported on 4 wt.% La_2_O_3_–Al_2_O_3_. This way, we were able to mimic the catalytic combustion process of methane in a real catalytic converter. By monitoring the voltage and current through the platinum heater in the absence and presence of methane, the thermal effects of catalytic combustion were indicated in the electric current variation, thanks to the positive temperature coefficient (TCR) of platinum. Thus, we were able to evaluate and finally discriminate between different synthetized catalysts.

## 2. Materials and Methods

### 2.1. Materials Preparation

All chemicals used in the preparation of catalytic materials come from commercial sources and have been used without the need for an additional purification step. The following chemical substances were used: cerium (III) nitrate hexahydrate 99.999%, manganese (II) nitrate hexahydrate 99.99%, lanthanum (III) nitrate hexahydrate 99.999%, aluminium nitrate nonahydrate 99.997%, citric acid ≥99.5% and a 25% solution of ammonia (to adjust the pH), all of which being purchased from Merck. As a support, commercially available Sasol Puralox TH100, an alumina containing 4 wt.% La_2_O_3_ with a specific surface area of 150 m^2^/g, was also used. In order to ensure the highest possible purity of the final materials, MiliQ deionized water was used in all the preparation steps.

The coprecipitation, incipient impregnation and citrate-based sol-gel were the three types of preparation methods employed in this study to obtain final catalysts with the CeO_2_:MnO_x_:(4 wt.% La_2_O_3_–Al_2_O_3_) molar ratio based on oxides of 7:3:10. The obtained materials were denoted as 7Ce3Mn/10LaAl_pp, 7Ce3Mn/10LaAl_imp and 7Ce3Mn/10LaAl_cit, with the front number corresponding to the molar ratio, while the termination representing the preparation procedures employed in these cases: coprecipitation, incipient impregnation and citrate-based sol-gel method, respectively.

#### 2.1.1. Coprecipitation Method

For the case of the coprecipitation method, in a first step, individual solutions were prepared containing the calculated amounts of metal precursors according to the molar ratio of 7:3:10. The required solutions were mixed and the pH of the resulting mixtures was adjusted to 9 by the addition of ammonia solution (percent concentration of 25%). The mixture was stirred overnight to ensure maturation of the material. The resulting solid material was filtered on a Buchner funnel and washed with deionized water. Drying of the materials was done in an oven at 60 °C for 1h (under vacuum), then the oven temperature was raised to 160 °C and left overnight (under vacuum). Finally, the materials were calcined at 500 °C for 6 h in air with a heating rate of 5 °C/min A 4 wt.% La_2_O_3_–Al_2_O_3_ support was also prepared by coprecipitation method as well, denoted further by LaAl_pp.

#### 2.1.2. Impregnation Method

For the case of incipient impregnation, in a first step the calculated amounts of metal precursors were dissolved in a required volume equal to the porous volume of deionized water necessary to completely wet the support. After dissolution of the precursors, the Puralox TH100 is added and homogenized well with a glass rod. The support was thus completely wetted without leaving solution outside the pores. The drying process plays an important role in the final properties of the catalytic material, special attention has been paid to the next stage. Therefore, the materials were slowly dried in the oven at 60 °C (under vacuum) for 1 h. The oven temperature is then raised to 80 °C (under vacuum) for another hour and was finally left at 160 °C (under vacuum) overnight. In all cases the heating rate was kept at 1 °C/min. Finally, the materials were calcined at 500 °C for 6 h in air with a heating rate of 5 °C/min.

#### 2.1.3. Citrate-Based Sol-Gel Method

Lastly, for the synthesis of the catalysts with the CeO_2_:MnO_x_:(4 wt.% La_2_O_3_–Al_2_O_3_) molar ratio based on oxides of 7:3:10, the citrate-based sol-gel method was used. Thus, in the first step, individual solutions containing the calculated amounts of metal precursors were prepared by dissolving the corresponding inorganic salts in deionized water and stirring at room temperature until homogeneous solutions were obtained. Later on, the citric acid complexing agent was added to each solution in calculated amounts to obtain a 1:2 weight ratio toward metal ions to citric acid but also an excess of 10%. The citrate mixtures were stirred at 50 °C for 2h. The water was evaporated using a rotavapor until gelling of the materials occurred. The obtained materials were dried under vacuum at 60 °C for 1 h and then the temperature was increased to 160 °C and kept overnight. The thermal stabilization process was performed at 500 °C for 6 h in air with a heating ramp of 5 °C/min. A 4 wt.% La_2_O_3_–Al_2_O_3_ support was also prepared by citrate method as well, denoted further as LaAl_cit.

### 2.2. Materials Characterization

Detailed features of all the samples were observed using a scanning electron microscope type GeminiSEM 500 (Carl Zeiss AG, Oberkochen, Germania) equipped with a Quantax XFlash 6/10 energy-dispersive spectrometry detector from Bruker (Billerica, MA, USA) for elemental analysis. Images of all samples were taken at the same magnification of 10,000 × and acceleration voltage of 1 kV using the InLens detector.

XPS measurements were performed using a Kratos Ultra DLD Setup spectrometer ( Kratos Analytical Ltd., Manchester, UK) using the Al–Kα (1486.74 eV) radiation produced by an X-ray source operating at a total power of 300 W (12.0 kV × 25 mA) and an approx. 1 × 10^−7^ Pa.

The temperature programmed reduction experiments in hydrogen (H_2_–TPR) were performed using a Porotec TPDRO 1100 device (Thermo Fisher Scientific Inc., Waltham, MA, USA). Prior to the reduction step, approximately 50 mg of sample was pre-treated for 1 h at 200 °C in a helium gas flow to ensure the surface cleaning, after which it was cooled down to room temperature also in a helium gas flow. Then a 5 vol. % H_2_–He mixture was passed over the sample with a flow rate of 50 mL/min and the temperature was linearly increased by 10 °C/min to 800 °C. The quantification of the hydrogen consumption during the reduction process was carried out by using the equipped thermal conductivity detector of the TPDRO device.

The X-ray diffraction measurements were performed using a Bruker-AXS D8 Advance diffractometer (Bruker Corporation, Billerica, MA, USA.) equipped with a LynxEye 1D detector and Cu-Kα (0.1541 nm) radiation source and a scintillation counter detector. The diffraction patterns were recorded over a 2θ range of 10–80° with a 0.01° step size and using a counting time of 1s per point. For the identification of the XRD phases present in the samples, the Powder Diffraction File from the International Centre for Diffraction Data (PDF-ICDD) was used.

The surface areas and pore size distribution of the as-prepared materials were determined by N_2_ adsorption–desorption isotherms at liquid N_2_ temperature (77 K) on a Micromeritics (ASAP 2020) analyser (Micromeritics Instrument Corporation, Norcross, GA, USA). Specific surface area and pore size distribution were calculated by Brunauer–Emmett–Teller (BET) formalism [23] and Barrett-Joyner-Halenda (BJH) method [24], respectively, while Langmuir surface area was determined by Langmuir formalism [25]. In order to efficiently remove the surface adsorbed residues, a degassing step at 150 °C for 4 h was employed.

#### 2.2.1. Preparation of the Self-Heated Micro-Converters

In order to proceed with the catalytic combustion investigations, catalytic micro-converters have been made on alumina substrate above which the catalyst was deposited by a drop coating method. More specific, the as received calcined powders based on CeMn/LaAl have been mixed with 1,2–propanediol as an organic binder and deposited over the platinum heater meander. The obtained catalytic layers were subjected to two heating stages. Within the first stage, the layers were kept at 60 °C for 18 h in order to settle the paste and to ensure optimum adhesion. As for the second heating stage, a programmed oven was used, applying the following protocol: 200–400 °C with 30 min at 50 °C step and 60 min at 450 °C. Consequently, the organic binder is removed leading to a homogeneous and porous catalytic layer formation. The as obtained catalytic micro-converters were labelled as follow: SENZ1 (7Ce3Mn/10LaAl_pp), SENZ2 (7Ce3Mn/10LaAl_cit) and SENZ3 (7Ce3Mn/10LaAl_imp).

**Figure 1:**
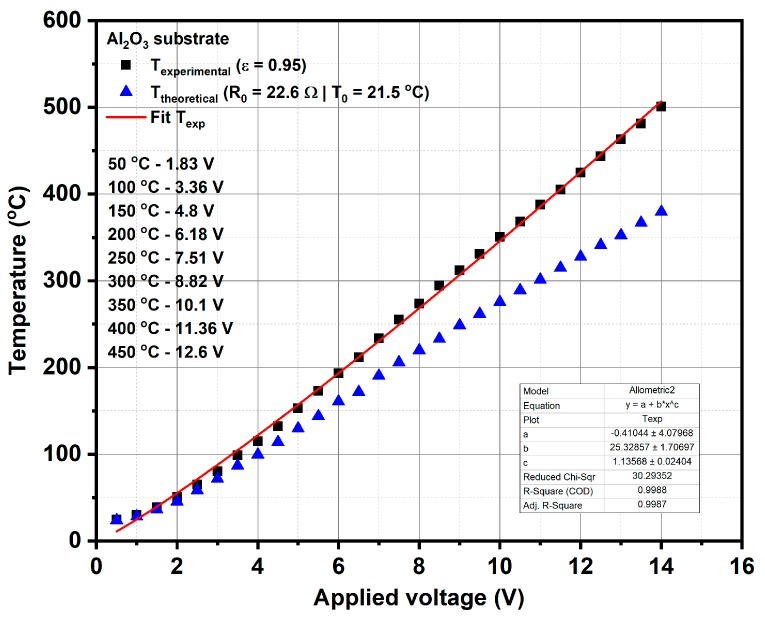
Temperature calibration curve with respect to the applied voltage; R_0_ is the electrical resistance at room temperature of 21.5 °C.

The calibration curve of the temperature of heater meander versus applied voltage was obtained using LumaSense IN-5L Plus pyrometer (LumaSense Technologies GmbH, Frankfurt, Germany) (see Figure 1), which provided the experimental temperature on the surface of micro-converter by considering a value of emissivity equal to 0.95 for the surface of the platinum meander. The calibration curve drawn with a continuous line, as shown in Figure 1, was obtained based on the linear fitting of the experimental results obtained by the pyrometer, at different applied voltages.

This calibration procedure is very important for subsequent catalytic combustion evaluations on our micro-converters in the presence of methane, because the operation temperature of these catalytic devices is controlled via the applied voltage on the heater side. This means that when the platinum heater is biased to a certain constant voltage, an electric current will flow through the platinum resistance and a temperature known via a previously-made calibration process will be obtained. In the presence of methane, due to the catalytic combustion of methane on the surface of the catalyst (which is an exothermic reaction), an increase in temperature will occur. A portion of this generated heat will be transferred to the platinum heater, which will increase its electrical resistance due to its +TCR and thus a decrease in the current flowing through the heater will be recorded. Such an electric current decrease (ΔI) through the platinum in the presence of methane in air, for the same applied voltage as in the case of humid air (without any methane added) will provide information about the presence of the catalytic combustion. Obviously, when the applied voltage is low and the temperature of the catalyst is too low, there is no catalytic process and the electric current through the platinum will not decrease even if methane is present at the surface of the catalyst.

### 2.3. CH_4_ Combustion Evaluation on the Self-Heated Catalytic Micro-Converter

**Figure 2:**
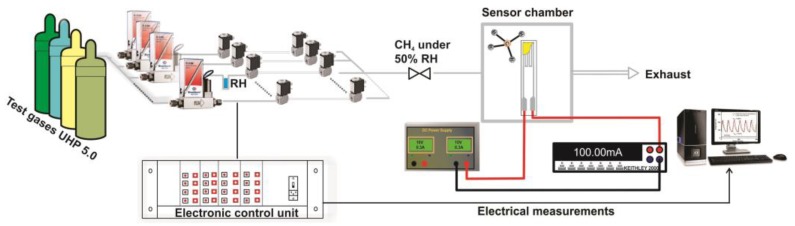
The setup for the evaluation of the catalytic properties of micro-converters exposed to CH_4_ in synthetic air with 50% relative humidity.

The catalytic properties of different catalytic micro-converters built with different CeMn/LaAl layers have been evaluated using a computer-controlled Gas Mixing System (GMS) consisting of a test chamber with 4 sensor sockets, a Keithley 2000-multimeter, DC regulated power supply source, mass flow controllers, vaporizers, valves and data acquisition cards. A dedicated software was used to control the gas mixture protocol and to record the electrical current changes passing through the heater (see Figure 2).

The catalytic micro-converters were exposed to 2500 ppm CH_4_ in synthetic air with 50% relative humidity (RH) specific for infield conditions and the flow rate was maintained constant at 200 cm^3^/min. The gas sensing performances have been acquired over a wide temperature range from 25 to 400 °C, obtained by applying a sequence of voltages from 1 V up to 11.36 V, on the heater as shown in the inset of Figure 1, for a period of 14 h. Each applied voltage was maintained for 1.75 h. During each time interval of 1.75 h, the catalytic micro-converter was first exposed to synthetic air with 50% RH for 1 h, followed by applying 2500 ppm of CH_4_ in synthetic air with 50% RH for 0.25 h and followed by exposure to the same synthetic air for 0.5 h, while the electric current was monitored and recorded continuously. In case of no catalytic combustion at a certain temperature, no current variation appears during the interval of time when CH_4_ is present in the synthetic air.

For the detection of light-off temperature of CH_4_ combustion, detectable by our specific approach, the experiment started at low applied voltages on the heater of catalytic micro-converter.

## 3. Results

### 3.1. Material Characterziation

#### 3.1.1. Textural Characterization

**Figure 3:**
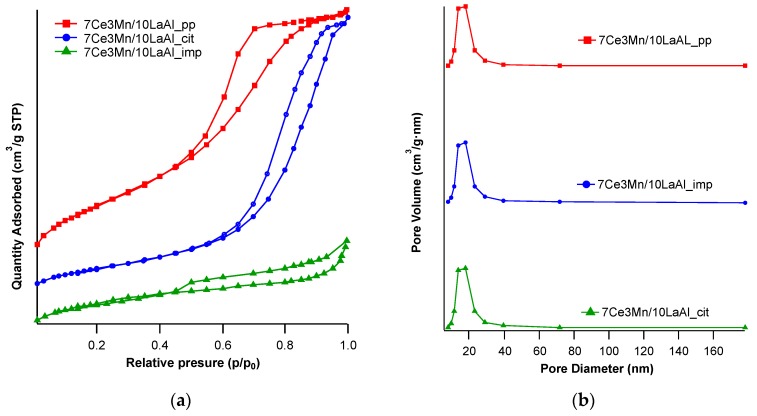
Textural characterization data for lanthanum-modified alumina supported cerium–manganese mixed oxides samples: (**a**) adsorption-desorption isotherms; (**b**) pore size distribution.

The nitrogen adsorption-desorption isotherms of the samples prepared by three different methods and calcined at 500 °C are presented in Figure 3a. The shapes of the isotherms are type IV according to IUPAC [26], characterized by a hysteresis loop at higher partial pressures associated with capillary condensation taking place in mesopores and the limiting uptake over a range of high P/P_0_.

The hysteresis of precipitated samples is type H2 characteristic to pores with narrow mouths (ink-bottle pores) or relatively uniform channel-like pores, while the samples prepared by the citrate and impregnation method present a type H4 hysteresis characteristic to narrow slit-like pores or particles with internal voids of irregular shape and broad size distribution. Indeed, the pore sizes distributions derived from the presented desorption isotherms for all samples show a broad pore size distribution with average value around 20 nm (Figure 3b) characteristic to mesopores materials.

Table 1 summarizes the surface areas, pore volumes and pore sizes for the samples prepared by different methods. The specific surfaces determined by BET formalism were 247 m^2^/g for 7Ce3Mn/10LaAl_pp sample and 105 m^2^/g for 7Ce3Mn/10LaAl_cit. The same trend was also observed for the samples prepared without Mn and Ce oxides, the precipitated sample—LaAl_pp—displays a surface area of 307 m^2^/g while the citrate sample—LaAl_cit—presents 127 m^2^/g. The pore volume was equal to 0.32 cm^3^/g for the precipitated sample and 0.11 cm^3^/g for the citrate sample, while the pore size was determined by the BJH formalism to be 5.2 and 4.9 nm for 7Ce3Mn/10LaAl_pp and 7Ce3Mn/10LaAl_cit, respectively.

The textural parameters of the sample prepared by citrate method are much more inferior to those of the materials prepared by coprecipitation, indicating a more pronounced sintering process, which probably generates higher particles sizes and therefore lower surface area.

For the catalysts prepared by incipient impregnation method, the specific surface area determined by BET formalism was 118 m^2^/g. Considering that the commercial support has a specific surface area of 150 m^2^/g and the specific surface decreases after impregnation with Mn and Ce oxides indicates that the deposited oxide particles on the surface partially block the pores. For all preparation method the specific surface loss is around 20%, which indicates that the Mn and Ce insertion has the same effect irrespective to the preparation method.

Is worth mentioning that all prepared samples present a contribution of a small fraction of micropores as is underlined by the difference between BET and Langmuir surface areas and depicted in Table 1.

To sum up, the specific surface areas of the various samples are in the following order: LaAl-pp > 7Ce3Mn/10LaAl_pp > 7Ce3Mn/10LaAl_imp >LaAl_cit> 7Ce3Mn/10LaAl_cit.

#### 3.1.2. XRD Analysis

**Figure 4:**
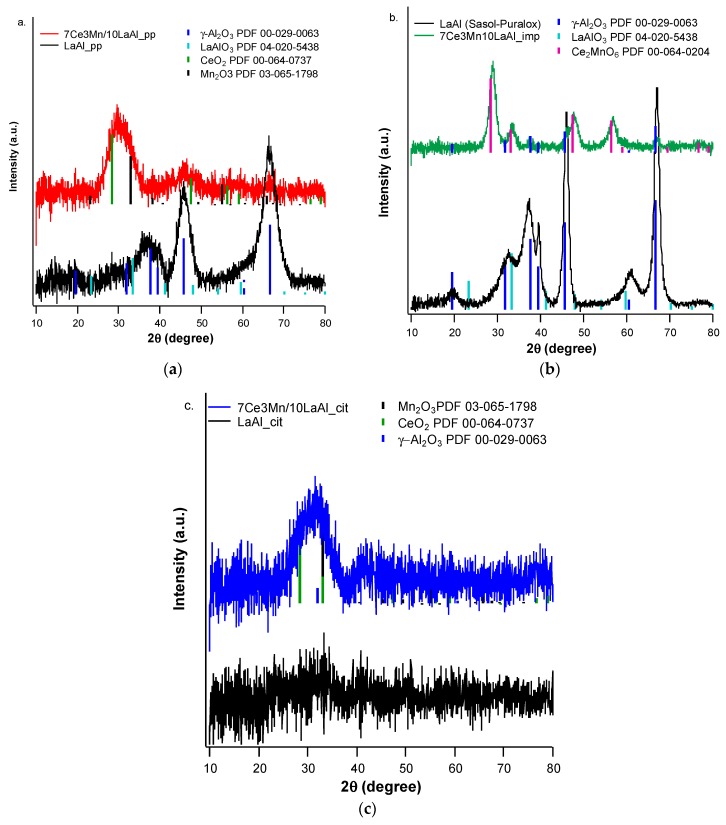
The X-ray diffraction (XRD) patterns of all CeMn/LaAl materials prepared: (**a**) coprecipitation method; (**b**) incipient impregnation of commercially available Sasol Puralox TH100 4 wt.% La_2_O_3_–Al_2_O_3_ and (**c**) citrate-based sol-gel method.

The powder X-ray diffraction patterns of all CeMn/LaAl materials prepared in this study are shown in Figure 4. The precipitated sample depicted in Figure 4a presents a low crystallinity and three main phases have been identified as: CeO_2_ or Ce_0.67_Mn_0.33_O_2_, which crystallizes on the same cubic network (PDF card 00-064-0737), Mn_2_O_3_, which also crystallizes in the cubic network (PDF card 03-0653-2798) and γ-Al_2_O_3_ in cubic structure (PDF card 00-029-0063). Thus, for the 7Ce3Mn/10LaAl_pp, CeO_2_ or Ce_0.67_Mn_0.33_O_2_ is found in the highest proportion, followed by Mn_2_O_3_ and γ-Al_2_O_3_. X-ray diffraction analysis was also performed on the 4 wt.% La_2_O_3_–Al_2_O_3_ support prepared by coprecipitation method. The XRD pattern of precipitate sample can be compared to the Sasol-Puralox TH100 commercial material (see Figure 4b). The material obtained through coprecipitation has the same phases as the commercial material (γ-Al_2_O_3_ cubic structure—PDF card 00-029-0063 and LaAlO_3_—PDF card 04-020-5438, approximately in the same proportions). However, a lower crystallinity of the coprecipitated material was observed, this being caused probably by a different nucleation processes occurring during the coprecipitation and also due to the lower calcination temperature used for our samples (500 °C) as compared with the one used for commercial sample (which was 550 °C). As a consequence, the coprecipitate sample possesses also higher surface area.

Figure 4b shows also the XRD pattern of 7Ce3Mn/10LaAl_imp sample. The material exhibits a low crystallinity, as well as samples prepared by coprecipitation and this is mainly due to the low calcination temperature (500 °C), which does not allow crystallization of the phases obtained at this temperature. For this sample, a mixed phase containing the impregnated metal oxides, that is, Ce_2_MnO_6_, with the diffraction lines 2θ of about 33, 47 and 57° was however identified. Also, specific diffraction lines of the γ-Al_2_O_3_ cubic structure (PDF card 00-029-0063) and the LaAlO_3_ (PDF card 04 -020-5438), phases present in the support (see Figure 4b) were observed.

The XRD pattern of 7Ce3Mn/10LaAl_cit sample is present in Figure 4c. It appears that, comparing with the XRD data of the materials described above, this preparation method leads to obtaining the weakest crystallized samples of this study. This time, the XRD pattern of the LaAl_cit support is no longer comparable with both commercial and coprecipitation derived supports, the common LaAlO_3_ phase (PDF 04-020-5438) being very difficult to be identified due to the weak crystallinity.

#### 3.1.3. Temperature Programmed Reduction Analysis

**Figure 5:**
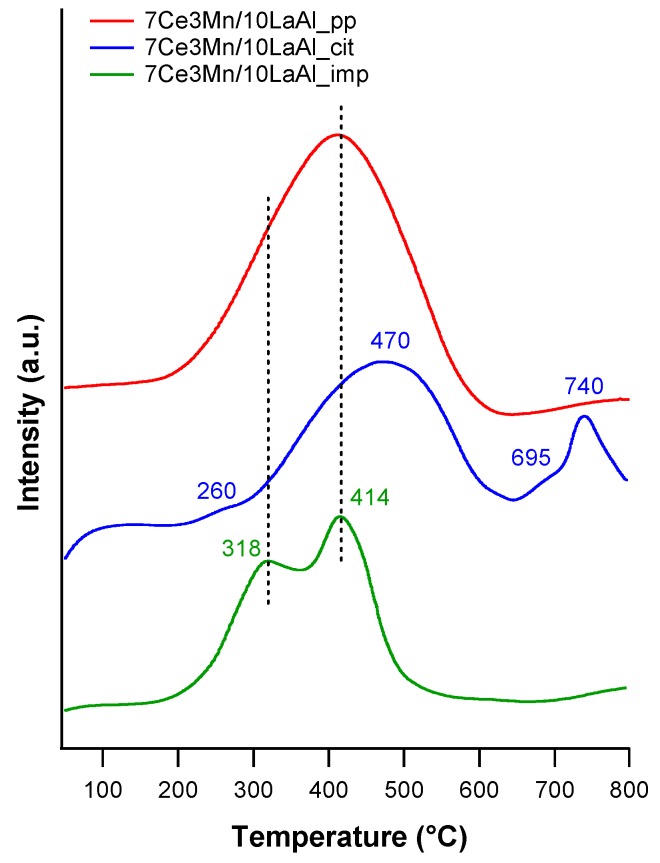
TPR–H_2_ profiles of the studied CeMn/LaAl samples.

In order to perceive the redox properties of CeMn/LaAl materials towards catalytic oxidation reactions, H_2_–TPR measurements were performed and the reduction profiles are exposed in Figure 5.

Therefore, to discern among the species involved into the H_2_ consumption, it is worth to mention that lanthanum-modified alumina has no hydrogen consumption until 800 °C and our finding is also supported by the literature [19]. In this way, the reduction peaks observed in the investigated temperature range can be ascribed only to the reduction of different types of cerium and manganese species. However, it is not a simple task to differentiate between the reduction of the two cations, since the reduction temperatures are quite the same, around 400–500 °C for the reduction of surface Ce^4+^ species and for the reduction of Mn^4+^ and Mn^3+^ species.

The reduction profile of CeMn/LaAl material synthesized by coprecipitation technique presents a broad reduction peak centred around 414 °C, which could be attributed to the reduction of MnO_2_ or Mn_2_O_3_ to Mn_3_O_4_ [19] as well to the reduction of surface Ce^4+^ [27] but it cannot be distinguished precisely. When cerium is present into a sample, usually two reduction peaks are observed, the low temperature peak characteristic to surface Ce^4+^ species and high temperature peak attributed to bulk Ce^4+^ species [27]. The fact that in this sample no reduction peak at high temperature is evidenced suggests that no bulk Ce^4+^ is reduced or, all reducible cerium is at the catalyst surface.

The reduction peaks for the CeMn/LaAl catalyst obtained by incipient impregnation are better defined and are attributed as follows: (i) the well-defined peak around 320 °C corresponds to the reduction of well dispersed small MnO_2_ particles through the alumina matrix [28]; (ii) the peak centred at 414 °C, which in the case of 7Ce3Mn/10LaAl_imp sample is shifted to lower temperature, is attributed to the reduction of MnO_2_/Mn_2_O_3_ species to Mn_3_O_4_ [19].

The H_2_–TPR profile of 3Ce7Mn/10LaAl material synthesized using citrate method (see Figure 5) contains four reduction peaks as follows: one, very weak, at 260 °C corresponding to isolated Mn^4+^ species, one at 470 °C assigned to the reduction of Mn_3_O_4_ to MnO and another two peaks at 695 °C and at 740 °C ascribed to the reduction of the bulk Ce^4+^ [29].

By comparing the three TPR profiles one can observe that, from the qualitative point of view, all samples present reduction peaks characteristic to the reduction of Mn^4+^ to Mn^2+/^Mn^3+^ and reduction of surface Ce^4+^ to Ce^3+^. The differences among the studied samples are related to the shift in the reduction temperature. The impregnated sample possesses small shifts towards lower reduction temperatures indicating a better reducibility associated to much more labile oxygen in the lattice. However, for the sample prepared by citrate method a reduction peak at high temperature characteristic to the reduction of bulk Ce^4+^ was observed. Summarizing, in our case the reducibility is highly dependent on the preparation method that probably has an influence over the interaction of the active species with the support.

#### 3.1.4. Scanning Electron Microscopy

**Figure 6:**
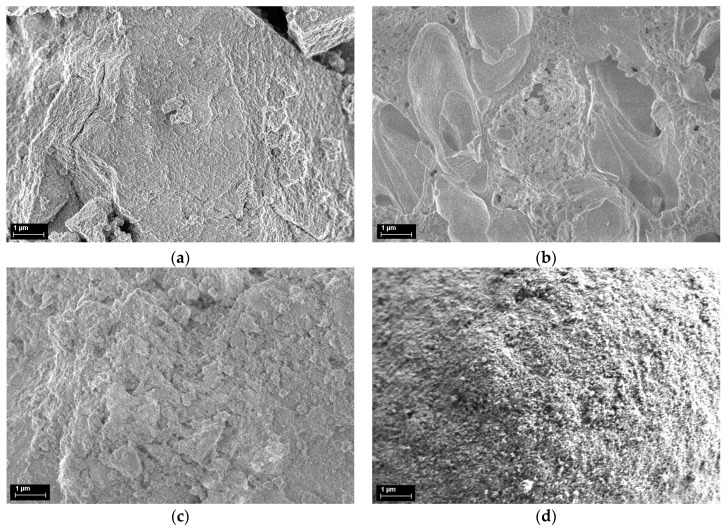
SEM images at 10,000× magnification of the 7Ce3Mn/10LaAl materials prepared by the following methods: (**a**) coprecipitation; (**b**) citrate-based sol-gel; (**c**) impregnation and (**d**) sample Sasol Puralox TH100.

Images of all samples are presented in Figure 6 and for comparison, were taken at the same magnification of 10,000× and acceleration voltage of 1 kV using the InLens detector.

SEM images show that sample fabricated by the coprecipitation method is agglomerated but presents fine pores on the surface (Figure 6a). The sample fabricated by the citrate sol-gel method shows porous surfaces as well as large size voids and indicate parts of the organic material that was not completely eliminated, in good agreement with surface area measurements (Figure 6b), for this sample being the smallest one. Finally, for the materials prepared by impregnation, the material is shaped as spheres with fine porous surfaces (Figure 6c) with the same morphology as the commercial support (Figure 6d) but higher diameters.

#### 3.1.5. X-Ray Photoelectron Spectroscopy

**Figure 7:**
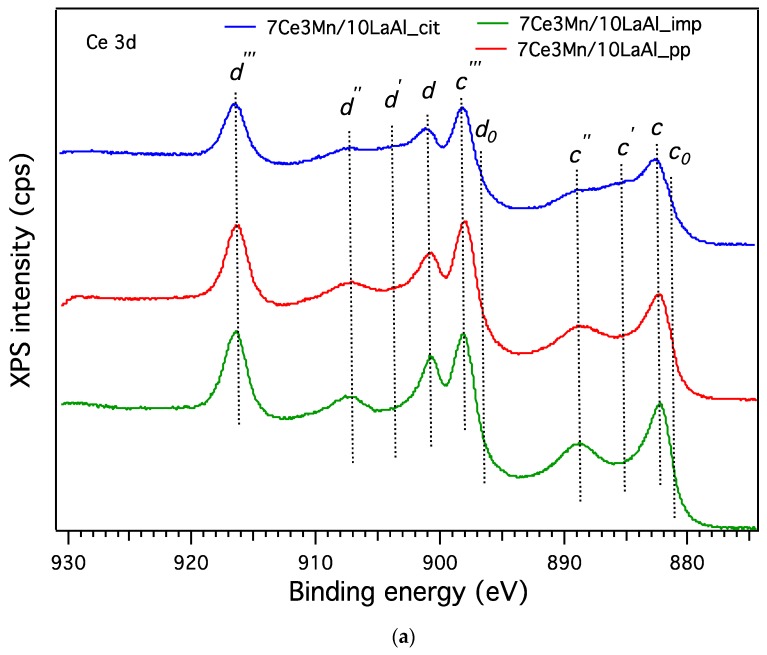

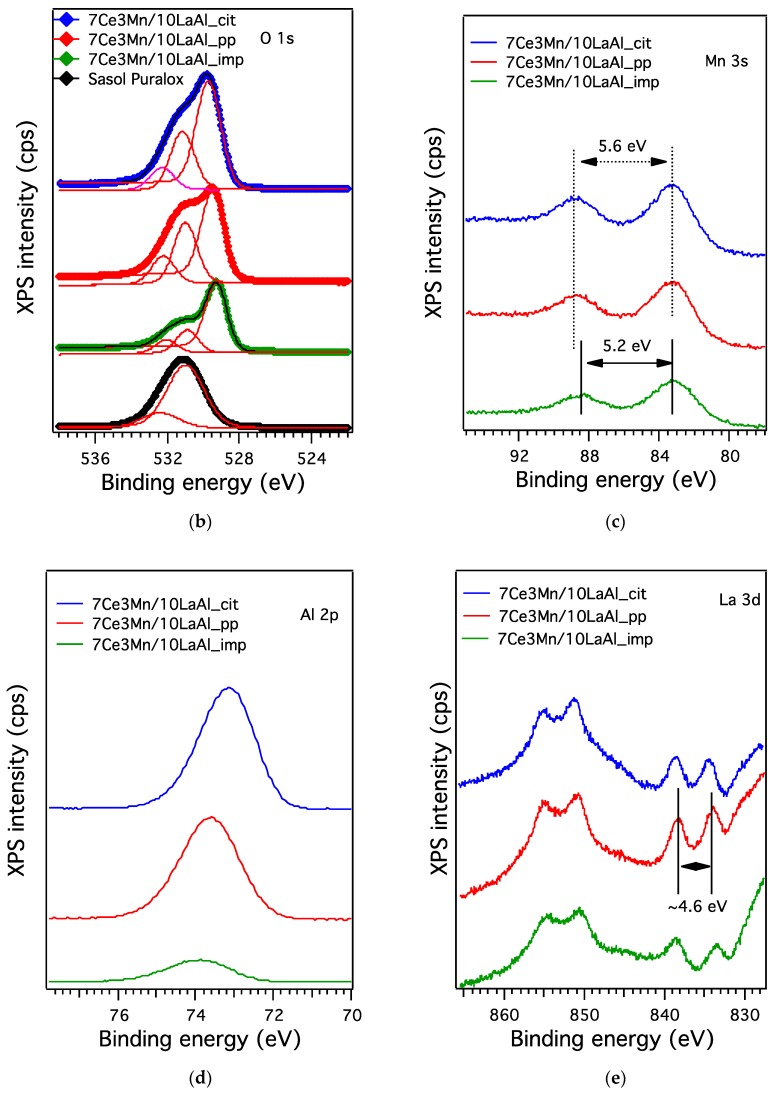

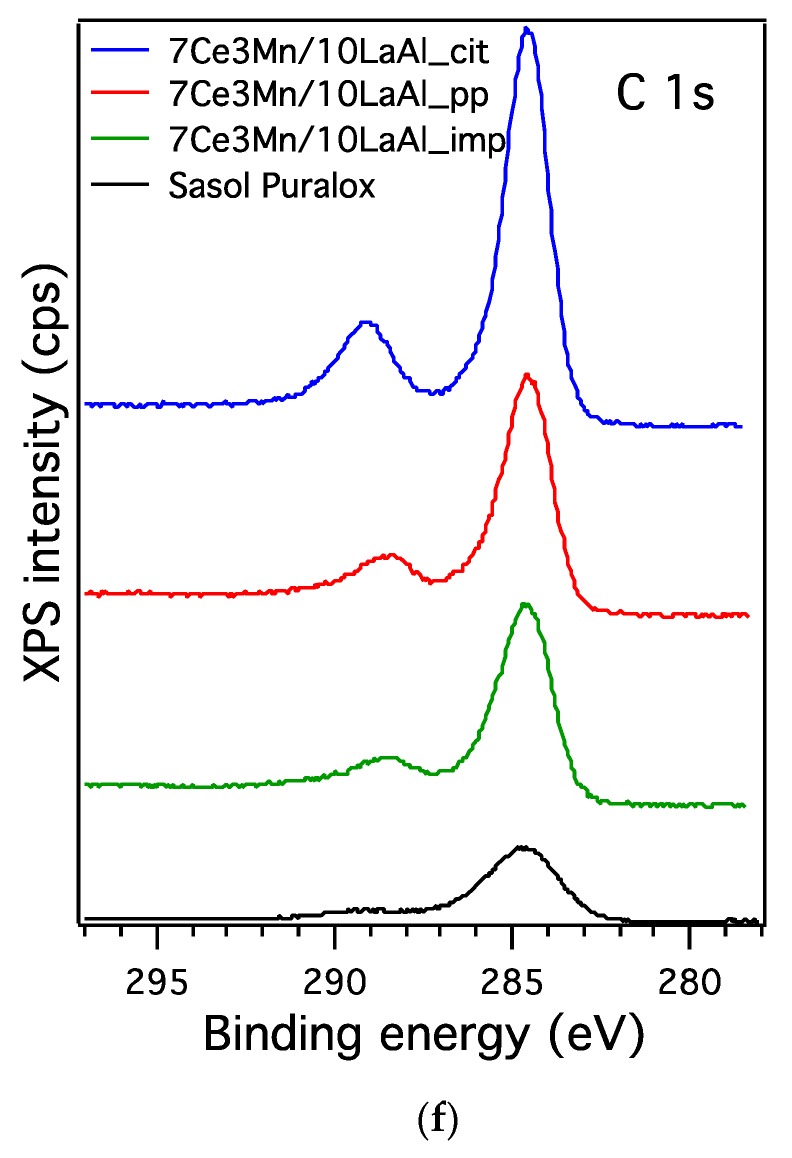
The high-resolution XP spectra of lanthanum-modified alumina supported cerium–manganese mixed oxides samples in the: (**a**) Ce 3d, (**b**) O 1s, (**c**) Mn 3s, (**d**) Al 2p, (**e**) La 3d and (**f**) C 1s high resolution regions.

XPS was used for the elemental qualitative and quantitative analysis of the components on the surface of all studied materials. For each sample, the general spectrum and their high resolution spectra were recorded and, for comparison reasons, were charge-corrected with respect to the C 1s peak located at 284.6 eV. From the general spectra, the presence of Ce, Mn, La, Al, C and O on the surface of all samples was identified. The high resolution spectra of the C 1s level (see Figure 7) are the same for all analysed samples, since carbon is present on their surface only as contaminant.

With respect to the high resolution spectra of Ce 3d (see Figure 7a), independently of the employed preparation procedure, the spectra indicate the presence of both oxidation states, namely Ce^3+^ and Ce^4+^, observation that is good agreement with XRD and H_2_–TPD data. According to the literature on Ce 3d spectra (Figure 7a), five spin-orbit split doublets were identified, denoted as c_0_/d_0_, c/d, c’/d’, c’’/d’’, c’’’/ d’’’ [30,31]. The c_0_/d_0_ and c’/d’ spin-orbit splits are assigned to Ce^3+^ oxidation state, while the other three spin-orbit splits are characteristic to Ce^4+^ state. What is interesting to note is that the XPS studies can confirm a higher presence of Ce^3+^ as compared to Ce^4+^ in the samples prepared by coprecipitation and citrate methods, probably induced by the generation of higher number of defects in bulk formed using these methods. In the sample prepared by impregnation, only a small amount of Ce^3+^ can be identified at the surface, the main species were indicated to be Ce^4+^.

The high resolution spectra of O 1s level (see Figure 7b) show broad and complicated peaks since on the surface there are at least four types of oxides with different non-equivalent oxygen ions. As expected, the high resolution spectrum of O 1s shows differences between the samples with respect to the ratios of the oxygen species present in the samples. Thus, for the support (4 wt.% La_2_O_3_–Al_2_O_3_) [31,32] two components at higher binding energy ~531.0 eV and ~531.2 eV were observed, while for all other three samples containing cerium and manganese, irrespective of the preparation method, a new component of oxygen, associated with Ce and Mn, at lower binding energies ~529.5 eV was identified [19,30,33]. The ratio between the oxygen associated with the Ce and Mn and the oxygen associated with the support varies depending on the synthesis procedures adopted in this study. Therefore, the impregnation method reveals at the surface a higher amount of oxygen related to the Ce and Mn against the oxygen linked to modified alumina with a ratio 1.8, while by citrate and coprecipitation method smaller ratios were identified (~1.3) and (~1), respectively. Consequently, according to XPS the impregnation method warrants a high amount of Ce and Mn species at the surface versus the citrate and coprecipitation methods, which allows probably a high and homogenous dispersion of the Ce and Mn species in bulk.

The high resolution spectra of Mn 3s (see Figure 7c) confirm, like in the case of cerium, dependently on the chosen preparation method, that different manganese oxides are formed on the surface of these materials. Therefore, in the high resolution spectrum of Mn 3s (Figure 7c) a difference of 5.6 eV between the two states is observed, which can be associated mainly to Mn^3+^ for the samples prepared by coprecipitation and citrate method. A smaller difference of 5.2 eV was observed in the sample prepared by impregnation method, denoting a higher amount of Mn^4+^ species in this case [34]. These results are again in good correlation with the XRD and H_2_–TPD data.

In Figure 7d can be observed that depending on the preparation method the binding energy peak of Al 2p is shifted to lower ones. In the sample prepared by impregnation, the Al 2p is found at ~74.0 eV corresponding to Al^3+^ from Al_2_O_3._ The observed shift of Al 2p to lower binding energies for the two other preparation methods (coprecipitation or citrate), where all the species are mixed together, is due probably to the differential charging of surface and bulk on such Al_2_O_3_ containing samples [35].

Figure 7e shows the high resolution spectra of the La 3d_3/2_ and 3d_5/2_, with maxima found at 833.9 and 850.8 eV respectively, which confirms that in the case of all samples the oxidation state of La is 3+. Also, the multiplet split of La 3d_5/2_ is 4.6 eV, corresponding to the oxide phase.

### 3.2. CH_4_ Catalytic Combustion Results

**Figure 8:**
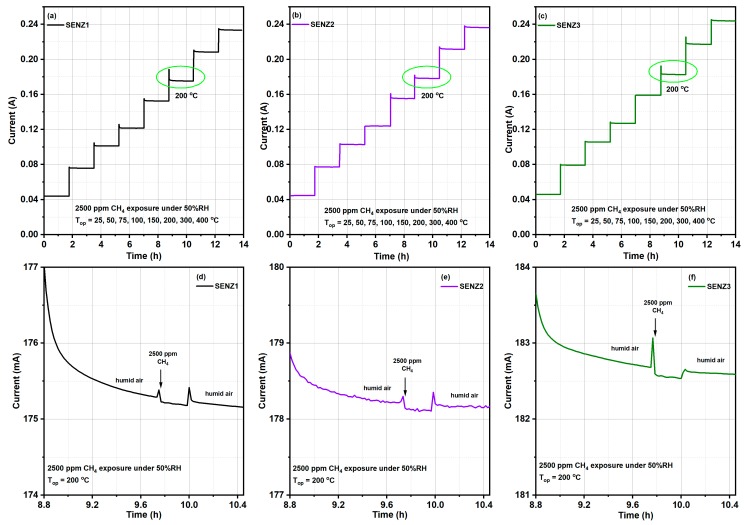
The electrical current dependence through the platinum heater of different catalytic micro-converters based on CeMn/LaAl catalysts with respect to the: (**a**–**c**) operating temperatures from 25 °C to 400 °C; (**d**–**f**) humid air-2500 ppm CH_4_ pulse-humid air @200 °C under 50% RH.

The catalytic behaviour of the SENZ1, SENZ2 and SENZ3 self-heated catalytic micro-converters was evaluated towards 2500 ppm CH_4_ under humid background conditions (50% RH), spanning the temperature range of 25–400 °C. Figure 8a–c show the electric current levels and associated temperatures from 25 °C up to 400 °C along the 14 h of micro-converters monitoring in different gaseous atmospheres, when the applied voltage increased from about 1 V at room temperature to 11.36 V at 400 °C, as described in the previous section. The overcurrent recorded in synthetic humid air at the change of each voltage level on the heater represent the electronic transient regime due to parasitic inductances and capacitances of the cables in the first tens of seconds, followed by the thermal transient regime for the stabilization of the microconverter temperature at each new level of applied voltage. Below temperatures equal to 200 °C (obtained for a constant voltage of 6.18 V applied on the heater) on the surface of micro-converter, very low current variation was detected when the 50% RH synthetic air was switched to 2500 ppm of CH_4_ in air for the best of the investigated devices, suggesting that a limited catalytic combustion was detectable by our method. The detailed variation of the electric current, before, during and after introduction of 2500 ppm of CH_4_ in the humid synthetic air is shown in Figure 8d–f, for the temperature of the surface of catalytic micro-converter equal to 200 °C. The electric current spikes at the change of gas composition in the testing chamber are related to this transient flow regime, even if the changes are made at constant total flow of gases. From Figure 8d–f, it is obvious that when 2500 ppm of CH_4_ was added to the humid synthetic air, an electric current decrease appeared with respect to previous and after regime due to an additional temperature increase of platinum resistance, by the heat provided by catalytic combustion of methane. The current variation (ΔI) is used for monitoring of the catalytic combustion of CH_4_ on different CeMn/LaAl catalysts.

Consequently, one can say that the catalytic micro-converters based on investigated CeMn/LaAl catalytic materials behave as true calorimetric sensors, namely, the CH_4_ is oxidized on the surface of the catalytic layer and the heat generated in the process is transferred to the Pt meander acting as a temperature sensor. More specific, the released heat raises the temperature of the heater, which is further transduced into a decrease of the electrical current.

**Figure 9:**
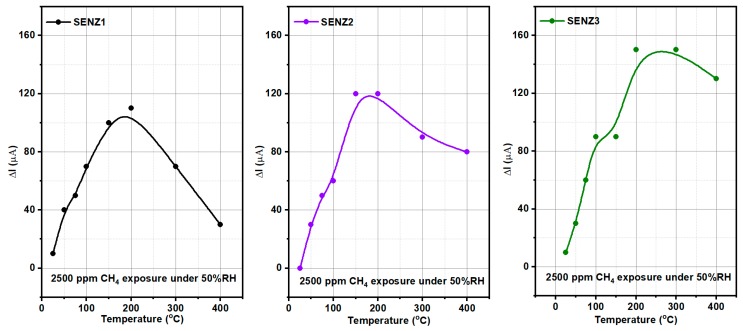
Conversion effects towards 2500 CH_4_ exposure in three different catalytic micro-converters with respect to the operating temperature, where SENZ1 is 7Ce3Mn/10LaAl_pp; SENZ2 is 7Ce3Mn/10LaAl_cit and SENZ3 is 7Ce3Mn/10LaAl_imp.

In Figure 9 the results of catalytic conversion of methane on different catalytic converters are presented, being expressed in terms of electrical current variation (ΔI) at each of temperature on the surface of catalytic converter, when 2500 ppm of methane was added to humid synthetic air. More specifically, the current difference ΔI = I_air_ − I_CH4_, where I_air_ represents the initial electrical current level (under the reference atmosphere containing 50% RH) and I_CH4_ represents the electrical current through the platinum heater/thermometer under the atmosphere containing 2500 ppm CH_4_.

The sensibility of the method used for the evaluation of the CH_4_ catalytic conversion, by ΔI recording, has made possible to identify a limited amount of CH_4_ molecules, which may be oxidized below 200 °C, while at 200 °C and above, higher amounts of oxidized CH_4_ molecules are obtained, as shown in Figure 9, within the limit of the experimental errors for reading the ΔI values from the Figure 8d–f. Thus, the results from Figure 9 can be used to evaluate the catalytic capabilities of different CeMn/LaAl. It appears that the catalytic micro-converter SENZ3 built with the catalysts made by incipient impregnation of commercial Puralox alumina and having the molar concentration CeO_2_:MnO_x_:(4 wt.% La_2_O_3_–Al_2_O_3_) = 7:3:10 has given the highest catalytic conversion of the CH_4_, while the same catalyst with the same stoichiometry but prepared citrate method (SENZ2), were placed on the a second position in this ranking process.

The stability (ζ) and reproducibility (Q) for the most sensitive catalytic micro-converter (calorimetric sensor SENZ3) was also investigated.

**Figure 10:**
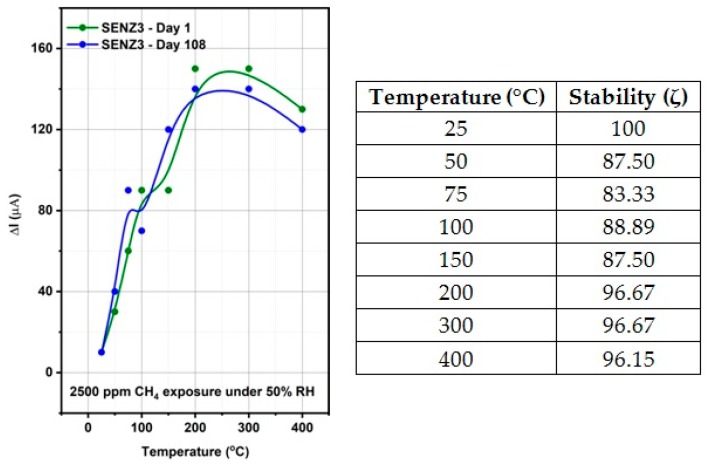
Stability of the conversion effects with respect to the SENZ3 (7Ce3Mn/10LaAl_imp) over 108 days.

The stability of a sensor describes the variation of the response over time (Figure 10) being quantitatively defined as:

(1) $${\zeta (p}_{i},\Delta t)=\left(\frac{\frac{1}{n}\sum_{i=1}^{n}x_{i}}{x_{\max}}{|}_{\Delta t}^{p_{i}}\right)\times 100$$

where *n* is the number of measurements, *x_max_* is the maximum sensor response value for a fixed operating temperature, *p_i_* is the present stimuli (2500 ppm CH_4_) and Δ*t* is the total measurement time.

For an ideal sensor, the stability is 100 while for all other sensors the stability values are between 0 and 100 [36]. In the case of calorimetric sensor SENZ3, the stability values are between 83.33 and 100.

**Figure 11:**
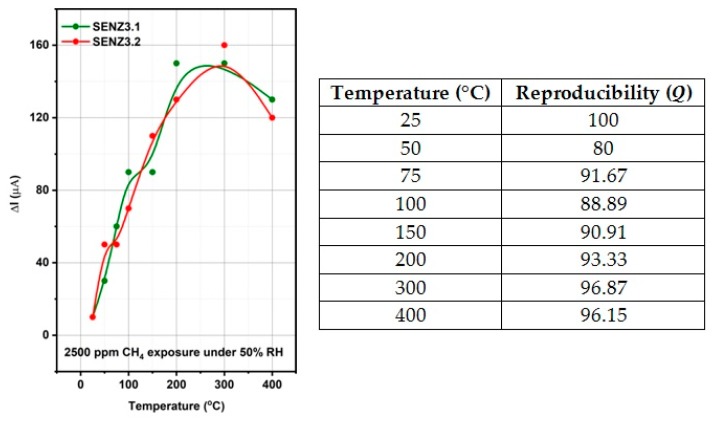
Reproducibility of the conversion effects for catalytic micro-converters of different batches (SENZ 3.1 and SENZ3.2) based on 7Ce3Mn/10LaAl_imp.

The reproducibility (Q) as similarity of individual catalytic micro-converters (SENZ3.1 and SENZ3.2) of different batches (Figure 11) has been also addressed:

(2) $$Q\;{(p}_{i})=\left(\frac{\frac{1}{n}\sum_{k=1}^{n}x_{k}}{x_{\max}}{|}^{p_{\mathrm{ij}}}\right)\times 100$$

where *n* is the number of characterized catalytic micro-converters, *x_k_* is the response of the sample k, *x_max_* is the maximum value of the response value for a fixed operating temperature and *p_i_* is the stimuli (2500 ppm CH_4_). The reproducibility values range from 0 (completely irreproducible) to 100 (perfectly reproducible catalytic micro-converters) [36]. In the case of calorimetric sensor SENZ3, the reproducibility values are between 80 and 100.

## 4. General Assessment

The catalytic properties of a material, as shown also by our own experimental results, are affected by the preparation step and due to its complexity is probably the most important step in the catalyst manufacture [37]. There are several factors, like geometry and the redox state of the metal cations that might alter the strength of the oxygen chemical bondings near the catalytic sites, thus affecting the catalytic activity. Also, to increase the rate of a catalytic reaction the catalyst must possess as many catalytic sites exposed to reactants and in this direction a special attention is dedicated to catalyst bulk versus surface properties modifications. Therefore, it is very important to find new ways to improve the preparation strategy to obtain more active catalysts, very well dispersed on a large surface area with enhanced stability.

Our different synthesis methods allowed us to investigate the effect of accessible active centre distribution on the surface of the support or intimately mixing of the catalyst at the bulk level with the support, as follows. In case of 7Ce3Mn/10LaAl_pp and 7Ce3Mn/10LaAl_cit catalysts, the precursors of the support were mixed with the precursors of the active species and therefore a bulk mixing between the active species and the support was obtained, while in the case of 7Ce3Mn/10LaAl_imp catalyst the active species were impregnated only on the surface of the commercial support, Puralox and therefore the active species are well dispersed on the surface, preserving an enough surface area (118 m^2^/g) with respect to initial value of the support (150 m^2^/g), as shown by our BET analysis (see Table 1).

In order to compare the behaviour of a bulk versus surface modification of alumina with Mn and Ce based oxides, a higher consideration was given to the interaction between Mn and Ce oxides and support. In the case of CeO_2_–MnO_x_ impregnated on commercial Puralox La-modified alumina support, our XRD results have indicated the presence of a mixed phase of Ce_2_MnO_6_ on the surface. Such a mixed phase may contribute locally to increased defects and further distortions of the atomic architecture of the catalytic sites and thus may further increase the catalytic activity of this type of supported catalyst.

The interaction of Mn and Ce oxides with alumina, on the surface or in bulk, is clearly demonstrated by the H_2_–TPR and XPS data. TPR-H_2_ analyses (Figure 5) evidenced the presence of the same type of reducible species for all samples. However, taking into account the shift in the reduction temperature and the lowest onset, it can be inferred that 7Ce3Mn/10LaAl_imp catalyst can be more active than the bulk ones, due to the higher mobility of the oxygen species and lower interaction of Mn and Ce oxides with alumina support. Moreover, more reducible sites on the CeMn/LaAl_imp catalyst surface facilitate the adsorption and activation of CH_4_ during the catalytic reaction and thus enhance its catalytic activity, in agreement with the theoretical predictions obtained by density functional theory (DFT) which showed a direct correlation between reaction energy for catalytic methane oxidation and surface reducibility of doped CeO_2_. [38]. From XPS data, higher ratio between O bonded to Mn and Ce and the O bonded to Al for the impregnated sample indicates that the oxygen species related to Mn and Ce are more active for CH_4_ combustion and have moderate strength (from H_2_–TPR data) with the support during the reaction. Thus, surface deposition of CeO_2_–MnO_x_ is much more desirable than the bulk one. Moreover, the data obtained from H_2_–TPR and XPS indicate the presence in higher amount of Mn^4+^ and Ce^4+^ for the impregnated sample, that promote the catalytic activity, as also indicated by other studies [39].

The presence of reducible Ce^4+^ and Mn^4+^ at the surface of impregnated the catalyst is an indication of Mars Van Krevelen mechanism, in which the oxidation of CH_4_ takes place using the oxygen from the lattice, with its rather labile chemical bonds to manganese and cerium cations, consecutively with the reduction of Ce^4+^ and Mn^4+^ to Ce^3+^ and Mn^3+^, respectively. Furthermore, the oxygen from the gaseous phase is used to reoxidize the surface to assure another catalytic cycle.

In agreement with the above material and catalytic properties of the developed catalysts, as depicted in Figure 9, the highest conversion rate was attained by the self-heated catalytic micro-converter denoted by SENZ3, obtained by impregnation of the CeO_2_–MnO_x_ catalyst on the commercial lanthanum-modified alumina support, Puralox. An additional explanation for this increased catalytic conversion of methane might be related to the increased diffusion of oxygen and CH_4_ toward catalytic sites due to higher pore sizes found for this impregnated catalyst, too (see Table 1) [40].

## 5. Conclusions

In this paper we studied noble metal-free catalysts consisting of Mn and Ce based oxides and lanthanum-modified alumina by three methods (coprecipitation, citrate-based sol-gel method and impregnation on a commercial 4 wt.% La_2_O_3_–Al_2_O_3_ support) for CH_4_ catalytic combustion. The experimental study aimed the elucidation of the role of the surface versus bulk incorporation of the CeO_2_–MnO_x_ catalyst on/in the support, respectively.

The self-heated catalytic micro-converters, which have been built for the functional testing of the catalysts, proved to be a sensitive test structure that identified very weak methane oxidation at temperatures below 200 °C.

The highest catalytic conversion of CH_4_ was obtained on micro-converts (SENZ3) where CeO_2_–MnO_x_ catalyst was impregnated on a commercial 4 wt.% La_2_O_3_–Al_2_O_3_ support (7Ce3Mn/10LaAl_imp). The heterogeneous combustion results with the maximum CH_4_ conversion for the CeO_2_–MnOx impregnated catalyst were correlated with the collective features such as: the highest pore sizes dimensions according with the BET investigations, the highest amount of Mn^4+^ or/and Ce^4+^ on the surface, presence of a mixed phase (Ce_2_MnO_6_) on the surface and a high reducibility of Mn^4+^ or/and Ce^4+^ species. The ranking of the catalytic activity of the three CeO_2_–MnOx catalysts evaluated by means of self-heated micro-converters was in direct correlation with the experimental surface reducibility results in hydrogen, as it was theoretically demonstrated in the prior art by DFT simulations.

This study has shown the superior catalytic activity of the CeO_2_–MnO_x_ catalyst impregnated on the surface of the support with respect to incorporation of the same catalyst in bulk.

By means of an extensive material characterization comprising XRD, BET, XPS and H_2_–TPR it was shown that surface modification of a commercial 4 wt.% La_2_O_3_–Al_2_O_3_ support with more accessible CeO_2_–MnO_x_ catalyst may be a promising direction for pushing further the research on complete oxidation of methane at lower temperatures, a major challenge of today’s research.

## 6. Patents

A patent application was recorded earlier in association with this paper.

## Figures and Tables

**Table 1:** Brunauer-Emmett-Teller (BET) and Langmuir surface areas, average pore size, pore volume and pore size of the samples prepared by three methods.

Sample	BET Surface Area (m^2^/g)	Langmuir Surface Area (m^2^/g)	Pore Volume (cm^3^/g)	Pore Size (nm)
7Ce3Mn/10LaAl_pp	247	345	0.32	5.2
7Ce3Mn/10LaAl_cit	105	146	0.11	4.9
7Ce3Mn/10LaAl_imp	118	161	0.8	9
Sasol-Puralox TH100	150	nd*	0.8	11

*nd = not determined.

## References

[1] Kim G. (1982). Ceria-Promoted Three-way Catalysts for Auto Exhaust Emission Control. Ind. Eng. Chem. Prod. Res. Dev..

[2] Zhang X., Caldeira K. (2015). Time scales and ratios of climate forcing due to thermal versus carbon dioxide emissions from fossil fuels. Geophys. Res. Lett..

[3] Porta P.D., Ferrario B., Cantaluppi A., Montalenti P., Fizioano A.G. (1975). Catalytic Cartridge. U.S. Patent.

[4] Wang C., Binder A., Toops T., Lauterbach J. (2017). Evaluation of MN and Sn-Modified Pd-Ce-Based Catalysts for Low-Temperature Diesel Exhaust Oxidation. Emiss. Control. Sci. Technol..

[5] Zhang P., Lu H., Zhou Y., Zhang L., Wu Z., Yang S., Shi H., Zhu Q., Chen Y., Dai S. (2015). Mesoporous MnCeOx solid solutions for low temperature and selective oxidation of hydrocarbons. Nat. Commun..

[6] Twigg M.V. (2007). Progress and future challenges in controlling automotive exhaust gas emissions. Appl. Catal. B.

[7] Di Pascoli S., Femia A., Luzzati T. (2001). Natural gas, cars and the environment. A (relatively) ‘clean’ and cheap fuel looking for users. Ecol. Econ..

[8] Choya A., De Rivas B., González-Velasco J.R., Gutiérrez-Ortiz J.I., López-Fonseca R. (2018). Oxidation of residual methane from VNG vehicles over Co_3_O_4_-based catalysts: Comparison among bulk, Al_2_O_3_-supported and Ce-doped catalysts. Appl. Catal. B.

[9] Hu L.H., Peng Q., Li Y.D. (2008). Selective synthesis of Co_3_O_4_ nanocrystals with different shapes and crystal plane effect on catalytic property for methane combustion. J. Am. Chem. Soc..

[10] Lelieveld J., Crutzen P.J. (1992). Indirect chemical effects of methane on climate warming. Nature.

[11] https://www.epa.gov/ghgemissions/understanding-global-warming-potentials.

[12] Russell A., Epling W.S. (2011). Diesel oxidation catalysts. Catal. Rev. Sci. Eng..

[13] Gandhi H.S., Piken A.G., Shelef M., Delosh R.G. (1976). Laboratory evaluation of three-way catalyst. SAE Trans..

[14] Pu Z., Liu Y., Zhou H., Huang W., Zheng Y., Li X. (2017). Catalytic combustion of lean methane at low temperature over ZrO_2_-modified Co_3_O_4_ catalysts. Appl. Surf. Sci..

[15] Peterson E.J., DeLaRiva A.T., Lin S., Johnson R.S., Guo H., Miller J.T., Kwak J.H., Peden C.H.F., Kiefer B., Allard L.F. (2014). Low-temperature carbon monoxide oxidation catalyzed by regenerable atomically dispersed palladium on alumina. Nat. Commun..

[16] Mukherjee D., Rao B.G., Reddy B.M. (2016). CO and soot oxidation activity of doped ceria: Influence of dopants. Appl. Catal. B.

[17] Skorodumova N.V., Simak S.I., Lundqvist B.I., Abrikosov I.A., Johansson B. (2002). Quantum Origin of the Oxygen Storage Capability of Ceria. Phys. Rev. Lett..

[18] KaSpar J., Fornasiero P., Graziani M. (1999). Use of CeO_2_-based oxides in the three-way catalysis. Catal. Today.

[19] Venkataswamy P., Jampaiah D., Lin F., Alxneit I., Reddy B.M. (2015). Structural properties of alumina supported Ce–Mn solid solutions and their markedly enhanced catalytic activity for CO oxidation. Appl. Surf. Sci..

[20] Wang Z., Shen G., Li J., Liu H., Wang Q., Chen Y. (2013). Catalytic removal of benzene over CeO_2_–MnOx composite oxides prepared by hydrothermal method. Appl. Catal. B.

[21] Shi L., Chu W., Qu F., Luo S. (2007). Low-temperature catalytic combustion of methane over MnO*_x_*–CeO_2_ mixed oxide catalysts: Effect of preparation method. Catal. Lett..

[22] Shi L., Chu W., Qu F., Li M. (2008). Catalytic performances for methane combustion of supported Mn-Ce mixed oxides. J. Rare Earth.

[23] Brunauer S., Emmett P.H., Teller E. (1938). Adsorption of Gases in Multimolecular Layers. J. Am. Chem. Soc..

[24] Barrett E.P., Joyner L.G., Halenda P.P. (1951). The Determination of Pore Volume and Area Distributions in Porous Substances. I. Computations from Nitrogen Isotherms. J. Am. Chem. Soc..

[25] Langmuir I. (1918). The adsorption of gases on plane surfaces of glass, mica and platinum. J. Am. Chem. Soc..

[26] Thommes M., Kaneko K., Neimark A.V., Olivier J.P., Rodriguez-Reinoso F., Rouquerol J., Sing K.S.W. (2015). Physisorption of gases, with special reference to the evaluation of surface area and pore size distribution (IUPAC Technical Report). Pure Appl. Chem..

[27] Xingyi W., Qian K., Dao L. (2009). Catalytic combustion of chlorobenzene over MnO_x_–CeO_2_ mixed oxide catalysts. Appl. Catal. B.

[28] Rotaru C.G., Postole G., Florea M., Matei-Rutkovska F., Pârvulescu V.I., Gelin P. (2015). Dry reforming of methane on ceria prepared by modified precipitation route. Appl. Catal. A.

[29] Zhou X., Lai X., Lin T., Feng J., Hou Z., Chen Y. (2018). Preparation of a monolith MnO_x_–CeO_2_/La–Al_2_O_3_ catalyst and its properties for catalytic oxidation of toluene. New J. Chem..

[30] Liwei J., Meiqing S., Jun W., Xia C., Jiaming W., Zhichang H. (2008). Redox behaviors and structural characteristics of Mn_0.1_Ce_0.9_O_x_ and Mn_0.1_Ce_0.6_Zr_0.3_O_x_. J. Rare Earths.

[31] Leinen D., Fernández A., Espinós J.P., Holgado J.P., González-Elipe A.R. (1993). An XPS study of the mixing effects induced by ion bombardment in composite oxides. Appl. Surf. Sci..

[32] Katta L., Kumar T.V., Durgasri D.N., Reddy B.M. (2012). Nanosized Ce_1−x_La_x_O_2−δ_/Al_2_O_3_ solid solutions for CO oxidation: Combined study of structural characteristics and catalytic evaluation. Catal. Today.

[33] Yan L., Kong L.B., Pan J.S., Ong C.K. (2003). Role of oxygen pressure in growth of CeAlO_x_ thin films on Si by pulsed laser deposition. J. Appl. Phys..

[34] Biesinger M.C., Payne B.P., Grosvenor A.P., Lau L.W.M., Gerson A.R., Smart R.S.C. (2011). Resolving surface chemical states in XPS analysis of first row transition metals, oxides and hydroxides: Cr, Mn, Fe, Co and Ni. Appl. Surf. Sci..

[35] Jacquemin M., Genet M.J., Gaigneaux E.M., Debecker D.P. (2013). Calibration of the X-Ray Photoelectron Spectroscopy Binding Energy Scale for the Characterization of Heterogenous Catalysts: Is Everything Really under Control?. ChemPhysChem.

[36] Weimar U. (2002). Gas Sensing with Tin Oxide: Elementary Steps and Signal Transduction. Habilitation Thesis.

[37] Hutchings G.J., Vedrine J.C., Baerns M. (2004). Heterogeneous Catalyst Preparation. Basic Principles in Applied Catalysis.

[38] Mayernick A.D., Janik M.J. (2008). Methane activation and oxygen vacancy formation over CeO_2_ and Zr, Pd substituted CeO_2_ surfaces. J. Phys. Chem. C.

[39] Liu C., Shi J.-W., Gao C., Niu C. (2016). Manganese oxide-based catalysts for low-temperature selective catalytic reduction of NO_x_ with NH_3_: A review. Appl. Catal. A.

[40] Cabot A., Vila A., Morante J.R. (2002). Analysis of the catalytic and electrical characteristics of different modified SnO_2_ layers for gas sensors. Sens. Actuators B.

